# Prise en charge des lésions traumatiques de membres au cours des missions de pacification au Nord du Mali

**DOI:** 10.11604/pamj.2018.30.295.16648

**Published:** 2018-08-30

**Authors:** Akloa Komlavi Ehlissou Kolima, Kombate Noufanangue Kanfitine, Lamboni Damessane, Gnandi-Piou Fare, Sama Hamza Dolès, Akpoto Messanvi Yaovi, Assenouwe Sarakawabalo, Assogba Komi, Abalo Anani

**Affiliations:** 1 Service de Chirurgie d’Orthopédie et Traumatologie du Centre Hospitalo-Universitaire Sylvanus Olympio, Lomé, Togo; 2 Service de Chirurgie d’Orthopédie et Traumatologie de l’Hôpital Saint-Jean de Dieu d’Afagnan, Lomé,Togo; 3 Service de Chirurgie Thoracique du Centre Hopitalo-Universitaire Sylvanus Olympio, Lomé, Togo; 4 Service de Chirurgie d’Orthopédie et Traumatologie du Centre Hospitalo-Universitaire de Kara, Lomé,Togo; 5 Service d’Anesthésie-Réanimation du Centre Hospital-Universitaire Sylvanus olympio, Lomé, Togo; 6 Service d’Anesthésie-Réanimation du Centre Hospitalo-Universitaire de Kara, Lomé, Togo; 7 Service de Neurologie du Centre Hospitalo-universitaire Campus, Lomé, Togo

**Keywords:** Prise en charge, lésions, membres, Mali, Treatment, lesions, limbs, Mali

## Abstract

En Février 2016, le camp de la Mission Multidimensionnelle Intégrée des Nations Unies pour la Stabilisation du Mali (MINUSMA) de Kidal a fait l'objet d'une attaque faisant de nombreux blessés et des morts. Le but de ce travail était de déterminer la nature des lésions des membres et décrire leur prise en charge. Il s'est agi d'une étude transversale descriptive portant sur les lésions des membres des victimes de l'attaque du complexe du camp de la MINUSMA de Kidal. Les paramètres étudiés étaient l'âge, le sexe, le délai d'admission, le siège des lésions et le traitement chirurgical d'urgence réalisé. Soixante blessés dont 57(95%) hommes et 3(5%) femmes ont été admis. L'âge moyen des patients était de 28,9 ans. Nous avons recensé 127 lésions sur les 60 blessés dont 109 (85,8%) siégeaient sur les membres. Apres l'examen clinique et radiologique, nous avons retrouvé 32 cas (29,3%) de délabrement musculo-cutanées, 11 cas (10,1%) de fractures qui étaient toutes ouvertes, 15 cas (13,7%) de brulûre et 8 cas (7,3%) de lésion vasculaire et nerveuse puis 7 cas d'entorse. Les blessés grave ont bénéficié d'une réanimation dès leur admission et 4 parmi eux ont été opérés en urgence. Les autres blessés ont été opérés une fois leur état hémodynamique stabilisé par les mesures de réanimations. Six patients (10%) ont été évacués vers un hôpital de niveau 3 pour la poursuite de la prise en charge. Quatre blessés (6,7%) sont décédés. Les lésions des membres sont fréquentes mais n'engagent pas directement le pronostic vital du blessé lorsqu'elles sont isolées. Leur principale hantise demeure l'infection et la gangrène qui peuvent aboutir à l'amputation.

## Abstract (en)

In February 2016, the camp of the United Nations Multidimensional Integrated Stabilization Mission in Mali (MINUSMA), in Kidal, was attacked and many people were wounded and killed. This study aims to determine the nature of extremity traumas and to describe their treatment. We conducted a descriptive cross-sectional study of extremity traumas in the victims of the attack on the camp of MINIUSMA in Kidal. The parameters studied were age, sex, admission delay, seat of the lesions and type of emergency surgical treatment performed. The study involved 60 wounded patients: 57(95%) men and 3(5%) women. The average age of patients was 28.9 years; 127 lesions were identified, of which 109 (85.8%) on the limbs. Clinical and radiological examinations revealed 32 (29.3%) musculocutaneous disruptions, 11 (10.1%) open fractures, 15 (13.7%) burns, 8 (7.3%) vascular and nervous injuries and 7 sprains. Severely wounded patients underwent resuscitation upon admission. Four of them underwent emergency surgery. The remaining wounded patients underwent surgery after restoration of hemodynamic stability with resuscitation. Six patients (10%) were transferred to a Level 3 Hospital for further treatment. Four wounded patients (6.7%) died. Extremity traumas are frequent but not life-threatening when isolated. They have a higher risk of infection and gangrene which can result in amputation.

## Introduction

Les conflits armés entrainent de nombreuses lésions dont la gravité dépend du type d'arme utilisé et de l'organe touché. Les techniques de sauvetage au combat enseignées aux combattants, l'évacuation rapide des blessés vers les structures de soins ont entrainé une diminution de la mortalité sur le champ de bataille [1]. L'équipement des combattants a été amélioré au fil des conflits avec des outils de protection individuelle faits de casque et de gilet par balle plus performant. Mais les membres sont toujours exposés et restent vulnérables aux projectiles. Leurs atteintes sont fréquentes et les séquelles fonctionnelles sont importantes [2]. La mortalité sur le champ de bataille a certes baissé mais, les prouesses de la technologie dans la fabrication des armes létales plus sophistiquées, engendrent davantage des lésions de plus en plus graves. Le chirurgien opérant sur un théâtre de guerre doit donc redéfinir ses modalités de prise en charge des blessés et une bonne connaissance des différents types de lésions rencontrés au cours de ces conflits s'avère nécessaire [1, 3]. En 2013 une Mission Multidimensionnelle Intégrée des Nations Unis pour la Stabilisation du Mali (MINUSMA) s'est installée en vue de pacifier le conflit entre l'armée régulière et les groupes djihadistes. Les forces de cette mission étaient régulièrement attaquées par ces groupes djihadistes [4]. C'est ainsi que le 12 Février 2016, le camp de la MINUSMA de Kidal a fait l'objet d'une attaque complexe avec des tirs de mortier et l'explosion d'une voiture kamikaze au sein du camp. La prise en charge des blessés s'est effectuée à l'hôpital de niveau 2 de Kidal. L'objectif de ce travail était de déterminer la nature des lésions des membres causées par cette attaque afin d'améliorer leur prise en charge et réduire les séquelles fonctionnelles.

## Méthodes

**Cadre d'étude:** L'hôpital de niveau 2 de Kidal est une structure de soins de référence dans le secteur Nord du Mali. Il est situé à l'intérieur du camp de la MINUSMA de Kidal et est mis sous la responsabilité du Togo. Sa principale mission est d'apporter les soins médicaux et chirurgicaux d'urgence aux combattants engagés dans la mission. Secondairement, il apporte au besoin une assistance médicale à la population civile de ce secteur. L'hôpital dispose d'une équipe médicale, d'une équipe d'évacuation aérienne, d'une antenne chirurgicale, d'une unité de radiologie, d'un laboratoire et d'un cabinet dentaire. Sa capacité d'accueil est de 20 lits. L'équipe chirurgicale est composée d'un chirurgien généraliste, d'un chirurgien orthopédiste, d'un médecin anesthésiste réanimateur et de six infirmiers (deux anesthésistes, deux instrumentistes, deux infirmiers urgentistes). Les ressources permettent d'effectuer 3 à 5 interventions chirurgicales par jour voire plus en cas d'attaque ou de sinistres. Les blessés étaient évacués des structures de niveau 1 vers l'hôpital de niveau 2 par l'équipe médicale des différents contingents. Notre équipe d'évacuation était sollicitée parfois pour aller chercher les blessés sur le terrain.

**Circonstances:** il s'est agi d'une attaque complexe organisée et coordonnée par les djihadistes. L'attaque avait débuté aux environs de 6 h 55 minutes par des tirs de mortiers sur le camp. Pendant que les mortiers s'abattaient sur le camp, un camion chargé de 800 kilogrammes d'explosif força l'entrée du camp et s'explosa à 300 mètres environ après son entrée. Les explosions des mortiers et de la voiture kamikaze ont entrainé de nombreuses victimes. Notre étude était une étude transversale descriptive portant sur les lésions traumatiques des membres des victimes de cette attaque du complexe du camp de la MINUSMA de Kidal. L'interrogatoire des blessés ou de leur entourage a permis de recueillir l'identité, l'âge, le sexe, et le délai d'admission. Le siège des lésions était établi après un examen clinique minutieux. La radiographie standard a permis de préciser le bilan des lésions osseuses. Les blessés instables étaient admis au bloc pour une prise en charge d'urgence. Après stabilisation de l'état hémodynamique, la décision de transfert du patient vers un niveau supérieur était prise en fonction des lésions du patient et de notre plateau technique.

## Résultats

**Figure 1:**
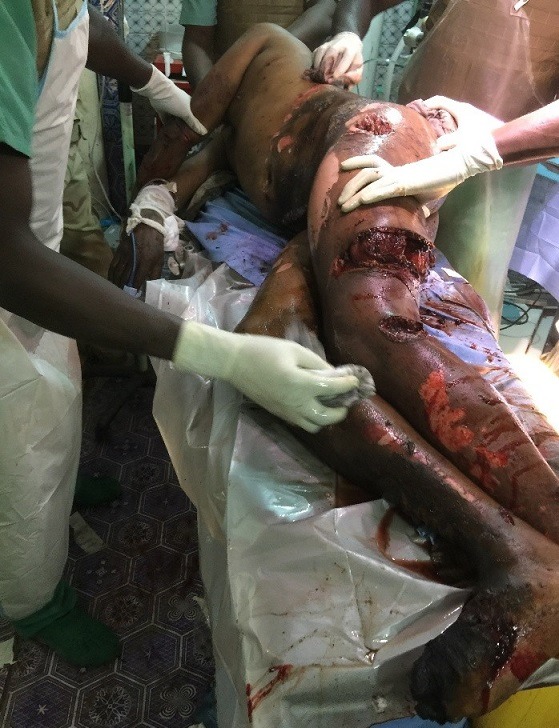
Militaire de 28 ans polytraumatisé avec une association lésionnelle

**Figure 2:**
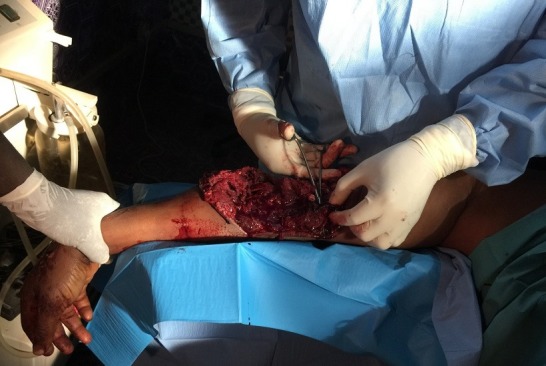
Militaire de 25 ans admis pour traumatisme ouvert du bras et de l'avant-bras type IIIc de Gustilo-Anderson

**Figure 3:**
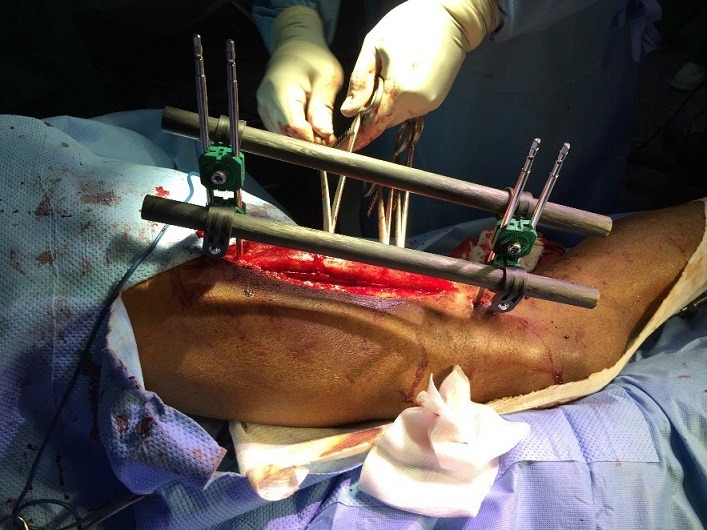
Fracture ouverte du Fémur chez un militaire de 27 ans, stabilisée par un fixateur externe

Nous avons reçu au décours de cette attaque 60 blessés dont 57(95%) hommes et 3(5%) femmes. L'âge moyen des patients était de 28,9 ans. Trente et un (51,7%) blessés ont été admis dans les heures suivant l'attaque, 11 patients (18,3%) ont été admis entre la deuxième et la sixième heure, et 22 autres patients (30%) ont consulté après la 6^ème^ heure. La plupart des blessés (58,3%) ont été amenés par les équipes médicales des différents contingents. Notre équipe d'évacuation a été sollicitée pour ramener 7 blessés (11,7%) grave à l'hôpital, et 18 autres patients (30%) ont consulté d'eux même. Nous avons recensé 127 lésions sur les 60 blessés dont 109 (85,8%) siégeaient sur les membres. Les 18 (4.2%) autres lésions étaient des lésions associées thoraco-abdominales, cranio-cérébrales, et otologiques. Les lésions siégeaient aux membres inférieurs dans 51,9% des cas et aux membres supérieurs dans 33,8%. Vingt-neuf (48,3%) patients présentaient une seule lésion, 17(28,3%) patients présentaient deux lésions et 14 (23,4%) patients présentaient trois lésions et plus. Aux membres inférieurs, Les lésions prédominaient à la jambe (16,5%) et à la cuisse (11,1%) alors qu'aux membres supérieurs l'épaule (11,1%) était le siège le plus concerné (Tableau 1). Le bilan clinique et radiologique a permis de noter 32 cas (29,3%) de délabrement musculo-cutanées, 11 cas (10,1%) de fractures qui étaient toutes ouvertes, 15 cas (13,7%) de brulûre (Figure 1), 8 cas (7,3%) de lésion vasculaire et nerveuse (Figure 2) et 7 cas d'entorse (Tableau 2). Concernant les lésions associées, nous avons retrouvé 4 cas d'hémothorax, 3 cas de plaies pénétrantes de l'abdomen et 3 cas de contusion pariétale thoraco-abdominale. Sept blessés graves ont bénéficié d'une réanimation dès leur admission et 4 parmi eux ont été opérés en urgence. Les autres blessés ont été opérés une fois leurs état hémodynamique stabilisés. Le geste réalisé était fonction du diagnostic retenu en préopératoire et des lésions observées au cours de l'intervention (Tableau 3). Ainsi nous avons procédé à une réparation tendineuse dans 9 cas et à une revascularisation dans 8 cas. Un fixateur externe a été posé chez 7 patients (Figure 3) et un débridement large a été réalisé pour les cas de délabrement musculo-cutanée. Nous avons réalisé une amputation de sauvetage chez 3 patients. Six (10%) patients ont été évacués vers un hôpital de niveau 3 pour une prise en charge appropriée. Les autres blessés ont été suivis dans notre hôpital jusqu'à la guérison. Quatre (6,7%) blessés ont succombés à leurs lésions associées qui étaient surtout thoraco-abdominale.

**Tableau 1:** Répartition selon le segment de membre atteint

	Effectif	Pourcentage (%)
Epaule	12	11,1
Bras	9	8,2
Coude	7	6,4
Avant- bras	7	6,4
Poignet	4	3,6
Main	4	3,6
Hanche	11	10,1
Cuisine	12	11,1
Genou	10	9,2
Jambe	18	16,5
Cheville	6	5,6
Pied	9	8,2
**Total**	109	100

**Tableau 2:** Répartition selon le type de lésions

	Effectif	Pourcentage (%)
Fracture	11	10,1
Délabrement Musculo-cutané	32	29,4
Contusion Musculaire	24	22,1
Brûlure	15	13,7
Entorse	7	6,4
Plaie + Lésions Tendineuse	9	8,3
Plaies + Lésions Vasculaires et Nerveuses	8	7.3
Amputation traumatique	3	2,7
**Total**	109	100

**Tableau 3:** Récapitulatif du traitement chirurgical en fonction de la lésion

Lésions	Traitement chirurgical
Fractures	Fixateur Externe ou Parage immobilisation
Délabrement Musculo-cutané	Parage
Lésions Tendineuses	Tendinorraphie
Lésions Vasculaires	Revascularisation
Amputation Traumatique	Régularisation du moignon
Brûlure	Fasciotomie, Décapage
Hémo-Pneumothorax	Drainage Thoracique
Plaie Pénétrante de l’abdomen	Laparotomie exploratrice

## Discussion

Cette étude présente un biais lié au faible échantillonnage mais les résultats mérites d'être comparés et discutés avec des données de la littérature. Soixante patients d'âge moyen de 28,9 ans ont été recensés. Le souffle de l'explosion et les fragments de métal propulsé sont à l'origine de ces lésions, souvent très contaminées. La proximité du sujet avec le centre de l'explosion détermine la gravité de la lésion [5]. Plus de la moitié des bléssés ont été admis à l'hôpital dans l'heure suivant l'attaque. Au cours de leur expérience à Kandahar, Khaplan *et al*. [6], ont trouvé un délai d'admission de 60-90 min après les traumatismes. Coupland *et al*. [7] n'ont reçu que 10% des blessés de guerre avant la 6^ème^ heure à l'hôpital de Kaboul, traduisant la difficulté d'accès de cet hôpital. L'hôpital de niveau 2 de Kidal étant situé dans le camp, le transfert des blessés s'est fait rapidement et dans un délai de moins de 6 heure pour la plupart des blessés. L'organisation du système de soins en différents échelons de niveau croissant permet de référer immédiatement les blessés nécessitant les soins chirurgicaux vers le niveau approprié et de préserver leur pronostic vital. La prise en charge des blessés dans la première heure était capitale pour leur survie (the golden hour des anglo-saxons) [1]. Les lésions de guerre sont particulièrement violentes et concernent les membres dans 70% des cas dont plus de 40% d'entre elles sont ouvertes et contaminées [8]. Nous avons enregistré 85,8% des lésions des membres dans notre étude dont 51,9% siégeaient aux membres inférieurs. Syed Awais *et al*. [9], ont retrouvé 78% de lésions des membres dans leur série. Akpoto et al ont retrouvé au cours de leur expériences sur les lésions des extrémités, 28,09% de délabrement musculo-cutanée; 20,23% de fracture ouvertes, 4,49% de lésions de brûlure. Nous avons recensé 13,7% de lésions de brulûre dans notre série qui serait surement lié à l'explosion de la voiture Kamikaze. Cette explosion produit davantage de lésions de brûlure que celle emmenant des tirs de roquettes, de mortiers et des engins explosifs improvisés [2, 9]. L'excision et le débridement venaient en tête de gestes chirurgicaux réalisés dans notre étude. La prise en charge des lésions des membres par arme à feu nécessite une excision large de tous les tissus nécrotiques ou de vitalité douteuse. Elle doit se de faire sous garrot afin d'être la plus large possible [10]. Seules les structures nobles sont conservées et recouvertes par du tissus sains de voisinage. Les plaies sont laissées ouvertes et leur fermeture ne se fait que secondairement à partir du cinquième jour après avoir éliminé tous les signes d'infection. Un second parage était fait lorsqu'on notait des signes d'infection et les plaies étaient laissées à nouveau ouvertes [11].

Les fractures sont traitées selon le principe du Damage contrôle orthopédique. Il consiste en une stabilisation provisoire, afin de contrôler l'hémorragie, de revasculariser le membre et de faire une excision des tissus nécrotiques tout en assurant une réanimation du patient. L'objectif étant de préserver le pronostic vital du patient puis l'évacuer vers un centre plus équipé où une stabilisation définitive par fixation externe ou interne pourra être effectuée selon l'état cutané [12-14]. La gestion des lésions par brûlure est difficile et longue surtout lorsqu'elles sont associées à d'autres lésions graves. En cas d'afflux massif de blessés brulés dans une zone de conflit avec des ressources limité, les soins sont portés d'abord aux brulés moins graves (brûlure de 20% - 70% de la surface corporelle brulée) au dépend des brulés graves. Les brulés de plus de 80% de la surface corporelle (brulés grave) requièrent plus de ressources et leur taux de mortalité est de plus de 50% dans ce contexte [2]. Nous avons évacué 10% de blessés vers une structure de niveau supérieur pour la poursuite de la prise en charge. Les autres ont été hospitalisés puis suivis à titre externe jusqu'à cicatrisation. La principale mission de l'hôpital de niveau 2 est d'assurer les soins médicaux et chirurgicaux afin de préserver le pronostic vital du blessé. Une fois le patient stabilisé sur le plan hémodynamique et la chirurgie d'urgence réalisée, le blessé était évacué vers un hôpital de niveau 3 où la prise en charge définitive était réalisée après un bilan paraclinique complet [15]. Le taux de mortalité enregistré au cours de cette attaque est faible comparé à celle de la littérature. Les travaux d'autopsie de Çelikel *et al* [16] réalisé au cours du conflit syrien en 2012 ont conclu que les sièges des lésions létales étaient par ordre de gravité: la tête, la nuque, le thorax et l'abdomen. Les lésions isolées des membres engageaient rarement à elles seules le pronostic vital des blessés.

## Conclusion

Les forces de la MINUSMA à Kidal sont délibérément agressées par les Djihadistes. Les segments de Membres sont le siège de lésions multiples. Bien qu'il soit le siège le plus fréquent, leurs lésions engagent peu le pronostic vital des blessés. Leur prise en charge est réalisé selon le principe du damage contrôle orthopédique. Une attention particulaire doit être accordée au débridement afin d'éviter les risques d'infection et de gangrène. La prise en charge définitive se fait secondairement. Cette stratégie thérapeutique a permis de réduire les séquelles fonctionnelles.

### Etat des connaissances actuelle sur le sujet

- La chirurgie traumatologique occupe une place importance au cours des conflits et des missions de pacification: le chirurgien est confronté à des lésions qui diffèrent de celles rencontrées en temps de paix;
- Malgré les mesures de protection individuelle qui sont de plus en plus performantes les membres sont toujours exposés et reste vulnérables aux projectiles: leurs atteintes sont fréquentes et les séquelles fonctionnelles sont importantes;
- La prise en charge de ces lésions des membres doit être correcte et bien codifiée afin de prévenir aux combattants des séquelles fonctionnelles qui sont parfois incompatibles avec la fonction militaire.

### Contribution de notre étude à la connaissance

- Il s'agit d'une première étude réalisée au Nord du Mali portant sur la prise en charge des lésions traumatiques des membres après une attaque kamikaze;
- La prise en charge a été faite dans une structure sanitaire isolée avec des ressources limitées;
- Les attaques Kamikazes se multipliant et survenant même en pleine zone urbaine, il nous semble opportun de partager nos résultats avec les collègues qui pourront être confrontés à la prise en charge des victimes de telles attaques.

## Conflits d’intérêts

Les auteurs ne déclarent aucun conflit d'intérêts.
